# 

**Published:** 1873-03

**Authors:** 


### EDITORIAL AND MISCELLANEOUS.

*The Georgia Medical Association.*

The annual meeting of the Georgia Medical Association will be held in the city of Atlanta on the 9th of April next. It will, we learn, be largely attended. We extend a warm welcome to our medical friends, and doubt not the meeting will be one of profit and pleasure to all.

*A Hint to Subscribers.*

We are constantly receiving letters requesting back-numbers of the *Georgia Medical Companion.* We regret to disappoint our friends, but, with the exception of a volume belonging to the editors, not a complete set can be had within our reach. The editions have long since been exhausted, and can only be supplied now by being reprinted. The circulation of the *Companion* increased so rapidly that, with a monthly issue of over a thousand copies, not one, of certain numbers, can be now found to supply the demands of our friends; and while regretting our inability to meet the wants of these friends, yet the statement will give the readers of the RECORD some idea of its rapid growth in the confidence of the profession. We have no doubt we shall exhaust, in a short time, the back-numbers of the RECORD, and hence, those desiring a complete volume should secure their copies at once. This advice we gave last year, and repeat it now for the benefit of all. Remember, the RECORD is published at $2.50 a-year, to be paid for in advance.

☞ We are making efforts to get up, from various sources, missing back-numbers. Let all our friends send for the numbers they wish, and we will exhaust every means to procure them.

*Communications.*

The following communications have been received: Medical Extracts: Practical and Philosophical—No. 1, by L. B. Anderson, M.D., of Virginia; The Prevalent Fever of Cherokee-Georgia, by Robert Battey, M.D.; Is This Woman Pregnant? by Robert Battey, M.D., of Georgia; Congestive Malarial Fever, by Benjamin Rhett, M.D., of South Carolina; Functional Diseases of the Eye—Hypermetropia, by Swan M. Burnett, M.D., Knoxville, Tennessee. Correspondence—Phagedenic Ulceration of Rectum, by Wm. R. Whitehead, M.D., Colorado Territory.

*List of Payments Received for the Record, Commencing with the January Number.*

Mississippi.—Dr. William M. Dunn, $2 50; Dr. L. M. Dampeer, 2 50; Dr. J. H. T. Stephenson, 2 50; Dr. J. J. Shelton, 2 50; Dr. G. W. Tribble, 2 50; Dr. R. H. C. Ferry, 2 50; Dr. John Gerdine, 2 50; Drs. Taylor & Price, 2 50; Dr. C. Y. Thompson, 2 50; Dr. G. W. Boothe, 2 50; Dr. A. G. Smythe, 2 50; Dr. R. F. Mathews, 2 50; Dr. J. W. Spillman, 2 50; Drs. T. H. Mayo and A. A. Lyon, 2 50; Dr. B. A. Vaughan, 2 50; Dr. F. H. Erwin, 2 50; Dr. J. G. Knox, 2 50; Dr. W. S. Chew, 2 50.

Georgia.—Dr. William Gaulding, 2 00; Dr. H. B. Lee, 2 50; Dr. C. C. Hart, 2 50; Dr. D. W. Hammond, 2 50; Dr. T. J. Jones, 2 00; Dr. J. E. G. Terrell, 2 50; Dr. J. B. Dillard, 2 50; Dr. Allen J. Belle, 2 00; Dr. F. L. Wisdom, 2 50; Dr. R. G. T. Halley, 2 50; Dr. J. W. Lambert, 2 50; Dr. T. M. Shaw, 2 50; Dr. E. C. Cocran, 2 50.

Alabama.—Dr. F. M. Rushing, 2 50; Dr. R. H. Lee, 2 50; Dr. William W. Wilkerson, 2 50; Dr. H. E. Hurst, 2 50; Dr. L. P. Hamner, 2 50; Dr. J. W. Andrews, 2 50; Dr. T. B. Fuller, 2 50; Dr. R. M. Brownlee, 2 50; Dr. Samuel H. Hill, 2 50.

Texas.—Drs. Sweatt & Kerr, 2 50; Dr. E. H. Watts, 2 50; Dr. J. H. Kerr, 2 50; Dr. T. J. Heard, 2 50; Dr. H. Shearer, 2 50; Dr. N. B. Butler, 2 50.

North Carolina.—Dr. Archibald Palmer, 2 50; Dr. Birdsong Burns, 2 50; Dr. Edward J. Ward, 2 50; Dr. A. H. Mann, 2 50.

South Carolina.—Dr. J. G. Smarr, 2 50; Dr. H. McNeil, 2 50; Dr. Robert McDaniel, 2 50.

Florida.—Dr. Robert Scott, 2 50; Dr. B. W. Taylor, balance, fifty cents.

Louisiana.—Dr. A. S. Helmick, 2 50; Dr. H. W. Traylor, 2 50.

Kentucky.—Dr. J. W. Williamson, 2 50.

Virginia.—Dr. J. A. Alexander, 2 00.

Michigan.—Dr. J. B. Payne, 2 50.

New York.—Dr. W. Gould, 2 50.

### The Southern Medical Record.

EDITED BY

T. S. POWELL, M.D.,

AND

W. T. GOLDSMITH, M.D.

ASSOCIATE EDITORS:

GEORGIA.

Robert Battey, M.D.
R. C. Word, M.D.
W. L. Kirkpatrick, M.D.
James A. Long, M.D.
W. L. M. Harris, M.D.
H. L Battle, M.D.
W. C. Musgrove, M D
L B. Bouchelle, M.D.
John C. Drake, M.D.
E. F. Knott, M.D.
Thomas Burdell, M.D.
E. H. W. Hunter, M.D.
V. S. Hitt, M.D.
J. E. Pope, M.D.
J. A. Hunnicutt, M.D.
A. H. Brantley, M.D.
J. J. Knott, M.D.
J. C. Wisdom, M.D.
W. W. Leake, M.D.

ALABAMA.

J. C. Knox, M.D.
Geo. F. Taylor, M.D.
J. B. Barnett, M.D.
S. M. Hogan, M.D.
D. S. Hopping, M.D.
J. T. Searcy, M.D.
S. F. Hill, M.D.

MISSISSIPPI.

C. B. Gallaway, M.D.
Edward Lea, M.D.
S. V. D. Hill, M.D.
W. B. Harvey, M.D.
J. W. M. Shattuck, M.D.
E. T. Henry, M.D.
T. P. Lockwood, M.D.
Robert Kells, M.D.

VIRGINIA.

Charles S. Lewis, M.D.
John H. Claiborne, M.D.
F. Horner, Jr., M.D.
R. J. Preston, M.D.
A. S. Payne, M.D.
J. E. Lockridge, M.D.
J. S. Wharton, M.D.
Wm. O. Owen, M.D.
Sam'l P. Ripley, M.D.
Benj. Blackford, M.D.
E. A. Drewry, M.D.
Rob B. Tunstall, M.D.
A. J. Terrell, M.D.
W. F. Barr, M.D.
L. B. Anderson, M D.
S. L. Jepson, M.D., W. Va.
J. M. Lazzell, M.D.

SOUTH CAROLINA.

E. T. McSwain, M.D.
G. M. Rivers, M.D.

TENNESSEE.

J. D. Tucker, M.D.
Swan M. Burnett, M.D.

TEXAS.

S. M. Welch, M.D.
T. J. Heard, M.D.
W. A. Heard, M.D.

ARKANSAS.

A. D. Hodge, M.D.
J. K. Hodge, M.D.

NORTH CAROLINA.

J. R. Hughes, M.D.
S. S. Satchwell, M.D.
A. B. Pierce, M.D.
H. Kelly, M.D.
Geo. A. Foote, M.D.
E. A. Anderson, M.D.
H. T. Bahnson, M.D.
Willis Alston, M.D.
Thos. F. Wood, M.D.
N. J. Pittman, M.D.

CORRESPONDING EDITORS:

J. M. Toner, M.D., Washington City, D. C.
John H. Weir, M.D., Illinois.
Thomas J Gallaher, M. D, Pennsylvania.
Ely Van DeWarker, M. D., New York.
Robert Robson, M D., Indiana.
C. C. F. Gay, M.D., New York,
*Surgeon of Buffalo General Hospital.*
W. T. Taylor, M.D , Kentucky.
J. Orne Green, Boston, Massachusets.
B. W. Stone, M.D., Kentucky,
*Assistant Physician Western Lunatic Asylum.*
James Tyson, M.D., Philadelphia. Pa.
W. W. Leen, M.D., Philadelphia.

M. H. Henry, M.D., New York,
*Surgeon to New York Dispensary, Department of Venerial and Skin Diseases.*
Wm. R. Whitehead, M.D., Colorado Territory.
A. Patton, M.D., Indiana.
Benjamin Howard, M.D., New York.
Chas Kearns, M.D., Kentucky.
S. C. Busey, M.D., Washington, D. C.
A. W. Hobson, M.D., Arkansas.
A. W. Calhoun, M.D., now of Berlin, Prussia.
T. Curtis Smith, Ohio.
Geo. Strawbridge, M.D., Philadelphia.

☞ *All Business Letters should be addressed to POWELL & GOLDSMITH, Post Office Box 527, Atlanta, Georgia.*

